# Metamorphosis in *Aurelia aurita* from polyp to medusa: assessing composition and metabolism throughout development

**DOI:** 10.1007/s42995-025-00329-2

**Published:** 2025-11-19

**Authors:** Vanesa Romero-Kutzner, Daniel R. Bondyale-Juez, Ico Martínez, Alicia Herrera, Theodore T. Packard, May Gómez

**Affiliations:** Marine Ecophysiology Group (EOMAR), IU-ECOAQUA, Universidad de Las Palmas de Gran Canaria, Canary Islands, Spain

**Keywords:** Excretion, Jellyfish, Lipid, Protein, Respiration, Strobilation

## Abstract

*Aurelia aurita* exhibits a triphasic life cycle involving metamorphosis, transitioning from sessile polyps to free-swimming ephyrae and eventually maturing into medusae. This metamorphic process is triggered by a reduction in temperature. In this investigation, we delve into the intricate changes in protein, lipid, and carbohydrate content, and examine alterations in respiratory and excretory metabolisms using both physiological and enzymatic methodologies. This study provides the first monitoring of these parameters. Observations at compositional and metabolic levels were conducted over 108 days in triplicate, with three simultaneous cultures maintained under identical conditions throughout the experiment. The findings reveal compositional changes, particularly in lipid content, one of the main sources of biological energy during metamorphosis. Additionally, a 20-day increase in water content from 89 to 99% occurred during the transition from strobila to metaephyra. Respiratory activity reduced by 76% during strobilation, due to the necessary temperature drop. Concurrently, excretory activity showed a more gradual increase in ammonium excretion during the planktonic stages once feeding resumed. These findings highlight the role of temperature-dependent triggers and metabolic shifts in facilitating energy storage among other functions. This knowledge may provide insights into the potential impacts of future environmental change on the entire lifecycle.

**Supplementary Information:**

The online version contains supplementary material available at 10.1007/s42995-025-00329-2.

## Introduction

Modern-day jellyfish are morphologically similar to their ancient cnidarian ancestors, which date back 635 million years ago to the beginning of the Ediacaran period, 94 million years before the Cambrian—a time when “the metazoa inherited the world” as described in Cloud and Glaes (1982). During this era, metagenesis began, and metamorphosis appears to have evolved up to eight times in metazoans (Hadfield 2000). Among metazoans, the ancient and complex metagenesis of cnidarians (Kraus et al. 2015; Marques and Collins 2004) has contributed to the success of this phylum (Pitt et al. 2013). This process involves an alternation between the sessile polyp form, attached to a hard substratum, and the motile, pulsating, medusoid form, capable of swimming through the ocean’s water column. The polyp form is regarded as the ancestral cnidarian adult body plan, with the medusa form considered a later secondary derivative (Brooks 1886; Collins 2002; Hadži 1953; Haeckel 1879; Korschelt and Heider 1893; Salvini-Plawen 1987).

Scyphozoans, a cnidarian class often referred to as “true jellyfish”, typically exhibit a triphasic life cycle that involves a motile planula larva. These bilaterally symmetrical larvae are produced during sexual reproduction by mature medusae. Upon attachment to a substrate, the larvae undergo a transformation into sessile polyps. Polyps engage in various forms of asexual reproduction, and some can metamorphose into pelagic medusae through a process known as strobilation. Strobilation begins with the segmentation of the polyp’s body through apical transversal constrictions. It culminates in the apical release of planktonic, star-shaped ephyrae that grow into medusae (Lucas et al. 2012). Processes of the life cycle are strongly influenced by temperature which is linked to medusae abundance (Wang et al. 2025). Therefore, anthropogenic changes could potentially affect the ecological role of medusae (Purcell et al. 2007). Moreover, several species of scyphozoans, after undergoing strobilation, are known to form massive proliferations, also called blooms, which can disrupt marine ecosystems. These blooms alter the structure of marine food webs by outcompeting other species for resources and playing relevant roles both as prey and as predators (Behrends and Schneider 1995; Lynam et al. 2006). Additionally, jellyfish blooms affect trophic pathways by shifting the flow of energy within the food web, and influence biogeochemical cycles, including nutrient cycling and the carbon pump (Pitt and Lucas 2014; Sweetman et al. 2016; Tinta et al. 2020).

With climate change and anthropogenic activities exerting an increasingly growing influence on marine environments, the precise impact of these factors on jellyfish populations remains a subject of ongoing debate (Pitt et al. 2018; Sanz-Martín et al. 2016). Nevertheless, many scientists argue that jellyfish are likely to play a key role in oceans in the future (Falkenhaug 2014; Lee et al. 2023; Richardson et al. 2009). This resilience stems from their ability to adapt to changing conditions, as they appear less impacted by anthropogenic activities that primarily affect their competitors. Given their evolutionary persistence over millions of years (Cartwright et al. 2007), understanding the biology and ecology of jellyfish is critical for predicting future dynamics in marine ecosystems and the broader ecological impacts of environmental change.

However, despite their resilience, jellyfish metamorphosis may prove more vulnerable to further environmental shifts, potentially disrupting their life cycle (Loveridge et al. 2021, 2024; Lowe et al. 2020). New evidence also suggests that transgenerational changes can affect jellyfish populations differently (Loveridge and Lucas 2023; Lu et al. 2020), adding complexity and unpredictability to how these populations will respond to climate change. While traditional studies on jellyfish ecophysiology have focused on a single life stage (Algueró-Muñiz et al. 2016; Chuard et al. 2019; Dong and Sun 2018; Klein et al. 2014), current research increasingly avoids such single-stage analyses. Instead, it seeks to understand the adaptive capacity of populations across multiple generations in the context of future ocean conditions (Aalto et al. 2020; Foo and Byrne 2016; Olguín-Jacobson et al. 2021; Pandori and Sorte 2019; Pineda et al. 2012; van der Lee et al. 2020). Moreover, given certain species’ capacity to form blooms, changes in environmental conditions during their life stages and transitional periods could significantly influence jellyfish populations and their effects on ecosystem functions and services. In this context, gaining insights into the responses of jellyfish populations to environmental changes and adaptations across generations requires understanding of their physiology and composition throughout their entire life cycle (Dong and Sun 2018; Gibbin et al. 2017; Goldstein and Steiner 2019).

The objective of this research is to study the metabolism of the scyphozoan *Aurelia aurita* throughout its life cycle under controlled laboratory conditions. *Aurelia aurita* is a well-studied and widespread (nearly cosmopolitan) species. The life cycle consists of five stages: planula, polyp, strobila, ephyra, and medusa. This study focuses on monitoring key physiological rates, metabolic enzymatic activities, compositional changes, and wet mass across these stages, including metaephyra (a transitional stage to medusa). Planula larvae were not included in this study. Specifically, respiration, ammonia excretion, and enzyme activities related to energy metabolism were measured during the transition from polyp to medusa.

We hypothesize that significant shifts in metabolic rates and biochemical composition occur throughout metamorphosis, reflecting the biological demands of each life stage. Specifically, we expect an increase in energy reserves, particularly lipids, during strobilation—a morphologically and structurally demanding phase. This increase is likely to be accompanied by elevated metabolic rates, which may indicate the physiological stress associated with these changes. As ephyrae develop into medusae, we predict further increases in metabolic rates, alongside an increase in water content (water*), driven by the growth and expansion of their gelatinous bodies. Furthermore, we expect metabolic rates to rise likely due to the energy required for swimming and buoyancy mechanisms, as well as the development of sexual reproduction capacity. These hypotheses aim to elucidate the dynamic physiological and biochemical changes occurring during the metamorphosis of *Aurelia aurita*, thereby enhancing our understanding of the biological processes underlying the transition from polyp to medusa. This study provides unprecedented insight, as it represents the first long-term study to follow the metabolic rates and biochemical composition transitions of *Aurelia aurita*.

## Materials and methods

### Culture and experimental design

**Fig. 1:**
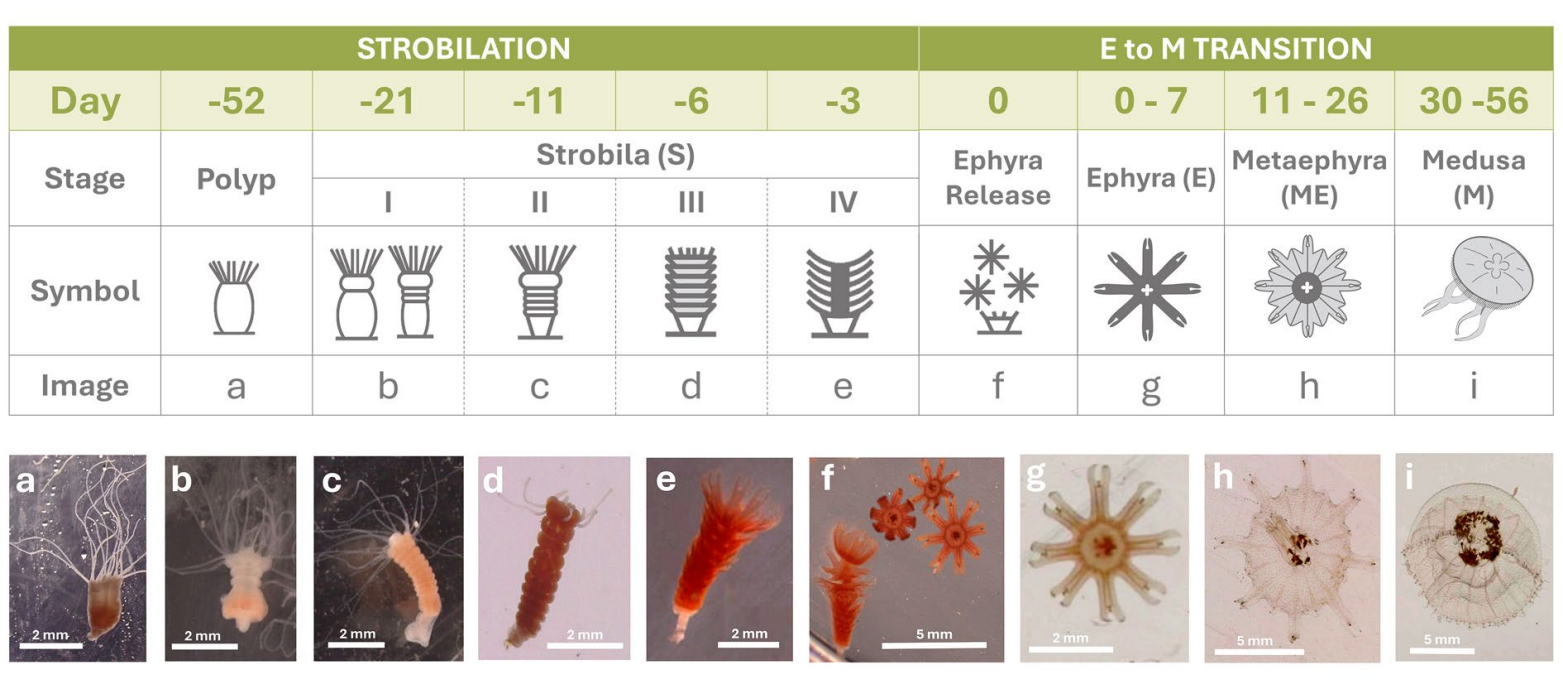
Day, stage name, symbols, and photos of the polyp, strobila stages measured, depiction of the ephyra release, and the planktonic stages in this study. Symbols modified from Kuniyoshi et al. (2012). Photos taken in this study using a stereoscopic microscope

Polyps, sexually produced from *A. aurita* planulae, were obtained from the Poema del Mar Aquarium (Gran Canaria, Spain). The stock culture was maintained in an 8 L tank at 23 ˚C, salinity 33, and pH 8.2 at the University of Las Palmas de Gran Canaria. They were fed once daily with *Artemia* sp. nauplii ad libitum. We define five main life stages: polyp, strobila (S), ephyra (E), metaephyra (ME), and medusa (M) (Fig. 1). To initiate the experiments, around 2000 polyps were placed in three 4.3 L aquaria and were acclimated for three weeks to a temperature of 20 ˚C (NUVE ES120 incubator).

The strobilation process was then induced by lowering the incubator temperature from 20 ˚C to 10 ˚C, reducing it by 1 ˚C per day (Kuniyoshi et al. 2012; Treible et al. 2018). In natural environments, temperature fluctuations of 10 ˚C over short periods are more likely in coastal waters and shallow seas where temperatures are closely coupled with the atmospheric conditions. The strobilation induction period lasted 52 days and four S stages (SI, SII, SIII and SIV, respectively) were included in this study according to Kuniyoshi et al. (2012) (Fig. 1). In Stage I of strobilation, the polyps begin to segment, and by Stage III, the body starts developing into distinct ephyra buds. Stage IV is the final stage before ephyrae release.

The results are presented in two sections: the first corresponds to the sessile life stages (Polyp and S) (day -52 to day 0), with day 0 marking the release of ephyrae, and the second covers the subsequent 56–day transition to the M life stage, resulting in a 108-day experiment (Fig. 1). Morphological changes during the life-stage transition are illustrated with symbols (Kuniyoshi et al. 2012) and photographs in Fig. 1, along with the corresponding experimental days when ingestion rates, wet mass (WM), biochemical content, and metabolic rates (both physiological and enzymatic) were measured. Polyp life stage measurements were taken before the start of metamorphosis.

During the experiment, *A. aurita* cultures were fed daily with 48 h-*Artemia* sp. nauplii (Hassan and Rahman 2016), and enriched with Selco (Easy DHA Selco, INVE Aquaculture). The aquaria were periodically checked every experimental day for physical and chemical changes. Specifically, parameters, such as temperature, pH (7.7 – 7.9), nitrate (NO_3_^−^) (0 – 10 mg L^−1^), nitrite (NO_2_^−^) (0 – 0.5 mg L^−1^), ammonium (NH_4_^+^) (0 – 0.2 mg L^−1^), and salinity (S) (32.5 – 33.5), were monitored. Around 10% of the seawater was replaced daily and completely changed on experimental days. Subsequently, the first ephyrae released from strobilae were measured on day 0. Then, ephyrae were transferred from the aquaria to 1.7 L Kreisel-tanks (specialized aquariums designed to maintain delicate marine organisms, such as jellyfish) to be cultured and measured during their transition to medusae.

### Experimental routine

This routine was always followed: first, the physical and chemical conditions of the water in the aquaria or Kreisels tanks were checked. Then the culture was fed for 1 h (Fernández-Urruzola et al. 2011; Purcell and Kremer 1983), with a known concentration of enriched 48-h-old *Artemia* sp*.* nauplii. After 1 h, the approximate food consumption was determined. Ingestion rates, per hour, were calculated by subtracting the final from the initial *Artemia* sp. nauplii concentrations (Supplementary Table S1). Simultaneously, the experimental organisms (polyps, strobilae, ephyrae, metaephyrae, or medusae) were separated from their respective culture tank and acclimated for 30 min in filtered seawater, as in Fernández-Urruzola et al. (2011). They were then placed carefully into respiration incubation flasks to determine oxygen (O_2_) consumption and NH_4_^+^ excretion. The number of organisms and the volume of the incubation flasks depended on the organism biomass: Polyps and strobilae incubations used 10 individuals in 5 mL, ephyra incubations used 15 individuals in 5 mL, metaephyra incubations used 10 individuals in 5 mL, and medusa incubations used between 3 and 5 individuals, depending on size, in 56 mL. The different-sized incubation flasks ensured an appropriate organism-biomass/seawater-volume ratio (Purcell et al. 2010). The incubation lasted ~ 1 h. A short incubation time minimizes enzymatic induction and repression, as well as starvation effects (Fernández-Urruzola et al. 2011). Furthermore, it should be within the time *A. aurita* takes to digest its prey (1–2.25 h, Uye and Shimauchi 2005). Also, NH_4_^+^ excretion rates are constant during a 2 h period (Nemazie et al. 1993; Purcell and Kremer 1983). Once the incubations were concluded, the WM was measured on an analytical balance. The samples were then stored at -80 ˚C for subsequent enzyme and biochemical analyses. This procedure was repeated, in triplicate, for each of the three aquaria/Kreisels cultures giving a total of nine samples on each measurement date.

### Biochemical components

The determination of protein (P), lipids (L), and carbohydrates (K) was based on the spectrophotometric methods proposed by Lowry et al. (1951), Bligh and Dyer (1959), and Dubois et al. (1956), respectively. Our modifications are given below. We approximated water content (water*) as WM – (P + L + K).

P analysis was based on Martínez et al. (2020). 100 µL of homogenate was mixed with 500 µL of the Rutter solution (Rutter 1967) with sodium dodecyl-sulfate sodium salt (SDS) (Markwell et al. 1978). After 10 min, 50 µL of Folin solution was added, mixed well, and left in darkness for 40 min. The absorbance was measured at 750 nm. Bovine serum albumin was used as the standard (0–500 µg mL^−1^).

L content, as an index of total lipids, was measured according to Barnes and Blackstock (1973), De Coen and Janssen (1997), Knight et al. (1972), and Marsh and Weinstein (1966). First, lipids were extracted following Bligh and Dyer (1959), using a chloroform-to-methanol-to-water ratio of 1:1:0.9 (this ratio takes into account the water in the sample). The mixture was centrifuged for 10 min at 2600 *g*, and 4 ˚C (Gerber et al. 2018). Then, 100 µL of the bottom phase was pipetted out and added to 500 µL of H_2_SO_4_ (95%). This solution was kept at 200 ˚C for 15 min, without mixing, and left to cool for 5 min, before it was mixed well. 40 µL of this solution was mixed with 1 mL of phospho-vanillin reagent and incubated for 15 min at 37 ˚C. The solution was then cooled for 5 min and its absorbance was measured at 525 nm. Commercial olive oil (0—4.8 mg mL^−1^) dissolved in chloroform was used as the standard.

K were determined following Dubois et al. (1956). 150 µL of a 5% phenol solution and 750 µL of a 95% concentrated sulphuric acid were added to 150 µL of homogenate. After 10 min, the solution was well mixed and incubated at 30 ˚C for another 10 min. Then, the sample was cooled to room temperature for 5 min and absorbance was read spectrophotometrically at 485 nm. Glucose (0–1.5 mg mL^−1^) dissolved in homogenate buffer solution served as the standard.

### Physiological respiration (R)

R was determined using O_2_-sensitive optodes (Fibox-4 system, PreSens) according to Lilley and Lombard (2015) and Bondyale-Juez et al. (2017). The O_2_-sensor was installed inside the transparent incubation vessels allowing non-invasive measurement with a fiber-optic probe. To minimize O_2_ concentration gradients, the incubation flasks were inverted three times before each measurement. R was determined as the change in O_2_ concentration during the incubation time (Δ[O_2_]/Δt). For control purposes, one flask was filled with filtered seawater without organisms (Osma et al. 2016). The incubations were conducted in darkness at 20 ˚C ± 0.2 ˚C for polyps, ephyrae, and medusae. Strobilae were incubated at 10 ˚C ± 0.2 ˚C, in accordance with the strobilation induction period.

### Physiological ammonium excretion (A)

A was determined using R incubations (Fernández-Urruzola et al. 2011; Solorzano 1969). An aliquot with 1 mL of seawater from the R incubation flasks was collected before commencing the R experiment and at its conclusion. The sample was mixed with 40 µL of 95% phenol solution, 40 µL of 0.5% sodium nitroprusside, and 100 µL of oxidizing solution (sodium hypochlorite and sodium citrate). These were added separately and meticulously mixed after each addition. A standard calibration curve (0–30 µmol L^−1^) was prepared in advance using NH_4_Cl (Sigma, A4514). After a reaction time of 1.5 h following the addition of reagents to the sample or standard, spectrophotometric analysis was performed at 340 nm (Cary100 UV–Vis Spectrophotometer, Agilent Technologies).

All glassware was cleaned the day before the experiment with diluted hydrochloric acid, then placed in a dishwasher with distilled water at 65 ˚C, stored in plastic bags and dried in an oven to prevent contamination until the analysis.

### Enzymatic measurements

After storage at -80 ˚C, *A. aurita* samples were homogenized in 0.1 mol L^−1^ phosphate buffer solution, pH 8.2 (Packard 1971; Packard and Christensen 2004; Tames-Espinosa et al. 2018), using an ice bath and an ultrasonic probe (Cole Parmer) with a Vibracell VCX 130 ultrasonic processor (Sonics) to release the enzymes. Subsequently, the homogenate was centrifuged at 4000 rpm (1500 g-force) for 10 min at 0–4 ˚C (Gómez et al. 1996). The supernatant of these centrifuged homogenates was used for enzymatic and biochemical analyses. The enzymatic analyses (ETS, IDH, and GDH) were conducted within 1 h of homogenization to avoid denaturation of the enzymes and loss of enzyme activity (Packard et al. 1974; Thuesen and Childress 1994). After these measurements were made, the remaining homogenate was stored at −20 ˚C for subsequent determination of biochemical composition. The temperature of the enzymatic assays corresponded to that of the physiological measurements, i.e., either 10 ºC or 20 ºC.

The respiratory ETS activity was measured according to Purcell et al. (2019). ETS enzymes are responsible for cellular respiration, and reflect the potential respiration (Φ) and the maximum R capacity (Packard 1985a, b). After centrifugation, 100 µL of the supernatant fluid was mixed in a cuvette with 300 µL of substrate solution containing NADH and NADPH (1.7 mmol L^−1^) in substrate buffer solution (at pH 8.6) and 100 µL of the indicator INT solution (2 mg INT mL^−1^ in double-distilled H_2_O). INT acts as an electron acceptor from the ETS before cytochrome oxidase, resulting in the production of an intense red formazan dye during the reaction. The formazan production was followed at 490 nm for 8 min; the slope of the time course reflects the ETS activity (Packard and Christensen 2004; Fig. 1). This rate (slope) is stoichiometrically related to ETS activity via the molar attenuation coefficient (12.8 mM^−1^ cm^−1^) measured for the formazan. The formazan production rate (µmol) is related by a factor of 2 to the ETS activity (µmol e^−1^) and by a factor of 0.5 to Φ in µmol O_2_ h^−1^ (Packard 1985a, b).

IDH activity was determined according to Tames-Espinosa et al. (2018). Following centrifugation, 100 µL of the sample-homogenate supernatant fluid was pipetted into a spectrophotometer cuvette. Then, 100 µL of 0.5 mM NADP^+^ (Sigma N0505) was added to a 300 µL mixture containing 3 mM DL-trisodium-isocitrate (Sigma l1252) and 6 mM MgCl_2_ (Panreac 131,396). These reagents were prepared in the same phosphate buffer solution (pH 8.2) mentioned earlier. NADP-IDH activity is the rate at which NADP^+^-dependent isoenzymes of IDH oxidize isocitrate and reduce NADP^+^ while producing CO_2_. NADPH and CO_2_ are related stoichiometrically, mole for mole, in the reaction:

$$ {\text{Isocitrate }} + {\text{ NADP}}^{ + } \leftrightarrow {\text{ alpha}} - {\text{ketoglutarate }} + {\text{ NADPH }} + {\text{ CO}}_{{2}} $$

IDH activity was monitored by the time-dependent NADPH production at 340 nm for 10 min. Respiratory potential CO_2_ production (IDH) was calculated by multiplying NADP-IDH activity by 3 (Roy and Packard 2001). This operation considers two other CO_2_-producing enzymes in the Krebs cycle, pyruvate dehydrogenase, and alpha-ketoglutarate dehydrogenase. The resulting IDH is expressed as µmol CO_2_ h^−1^.

GDH activity was measured according to Bidigare and King (1981) and Fernández-Urruzola et al. (2011). Post-centrifugation, 40 µL of the supernatant fluid of the sample-homogenate was pipetted into a 96-well microplate. Subsequently, 60 µL of a 1.2 mM NAD^+^ and 50 µL of 2 mM ADP, along with 100 µL of 10 mM sodium glutamate, prepared in the substrate buffer solution (pH 8.6), were added. The fluorescence increase, resulting from NADH produced by the reduction of NAD^+^, was followed fluorometrically at 360 nm excitation and 460 nm emission wavelengths for 10 min (Microplate reader, FLUOStarOmega, BMG Labtech) at 20 ˚C (Fernández-Urruzola et al. 2011) (as previously mentioned, strobilae were analyzed at 10 ˚C). This was converted to activity (µmol NH_4_^+^ per time) based on a standard curve, which was prepared from pure GDH (1.4.1.3) extracted from bovine liver (Sigma G2626). The curve ranged from 0.039–0 international units (U) of GDH activity mL^−1^, where one U equals the quantity of enzyme capable of converting 1 µmol NAD^+^ to NADH, and 1 µmol NADH converts to 1 µmol NH_4_^+^. Hereinafter, GDH activity is expressed as µmol NH_4_^+^ h^−1^.

### Statistical analyses

Significant differences between biochemical composition, respiration rates, excretion rates, and ratios through polyp and ephyrae to medusa transition were analyzed. Normality was evaluated by the Shapiro–Wilk test and variance homogeneity was tested by the Fligner–Killeen or Bartlett tests. In cases where the Fligner–Killeen test was employed and significant differences were observed, further analysis was conducted using the Kruskal–Wallis test. Pairwise comparisons were carried out using the Conover–Iman test to identify specific differences between unequal-sized groups. Where the Bartlett test was used, the data were analyzed using ANOVA, and significant differences were subsequently confirmed through the Tukey-HSD post hoc test (Conover and Iman 1981; Shingala and Rajyaguru 2015). All statistical analyses were performed using the R program (R Core Team 2017).

We decided not to change the incubation temperature (either 20 °C or 10 °C) during metabolic rate measurements (physiological and enzymatic), as strobilation is generally triggered by a decrease in temperature in natural conditions (Sukhoputova and Kraus 2017). Consequently, the strobila stage was excluded from the statistical analysis due to resulting 10 °C temperature difference between the strobila culture and the other life stages.

## Results

Benthic life stage (polyp) measurements started on day –52 (52 days before ephyrae release), and strobilae (S) were measured in four stages (day – 21 to day –3). Scyphozoan planktonic life forms were followed from ephyrae (E) release (day 0) until day 56 (Fig. 1). Strobilae stages (Fig. 1) were measured on day –21 (Stage I), day –11 (Stage II), day –6 (Stage III), and day –3 (Stage IV).

The data can be divided into two distinct transformation periods: strobilation (from polyp to E release) and the E-to-medusa (M) transition (Fig. 1). Strobilation lasted approximately 52 days, while E-to-M transition lasted 30 days. By day 30, the organisms appeared to be fully developed medusae. Afterward, the medusae were cultured for 26 more days during which they continued to exhibit growth.

Ingestion patterns of enriched *Artemia* nauplii were systematically observed. During the strobilation phase, a slight reduction in prey consumption was detected in S compared to the initial polyp stage. Notably, Stage IV strobilae and ephyrae exhibited minimal to negligible ingestion activity until day 4 post-release. Following this period, ingestion rates increased exponentially during the transition to the medusa stage (Supplementary Table S1).

**Fig. 2:**
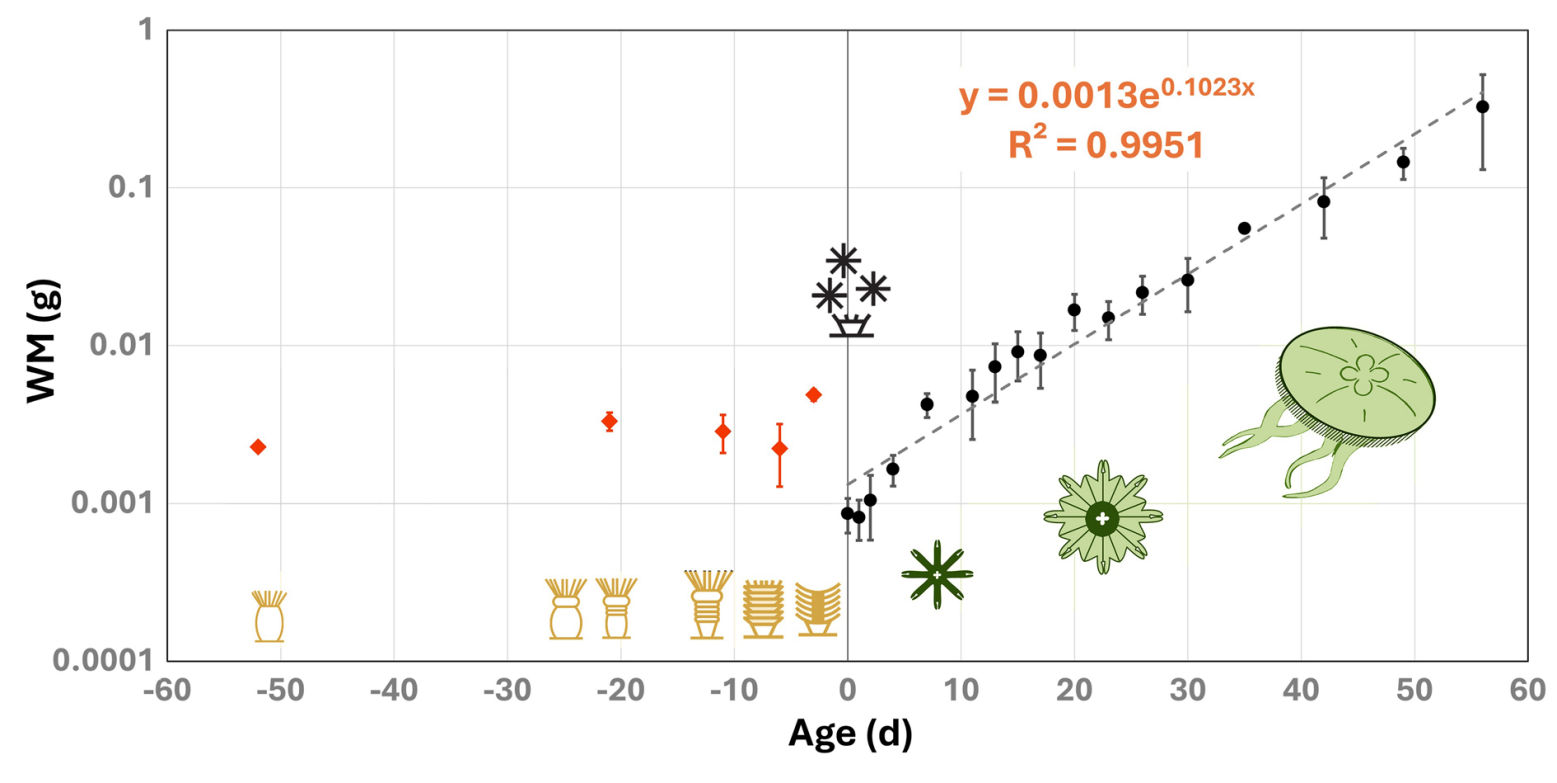
Changes in diameter and WM during the strobilation and transition from ephyra to medusa. Icons show the stage of the organisms and day 0 corresponds to ephyra release. WM in grams, exponential regression with time, y-axis in log scale

**Fig. 3:**
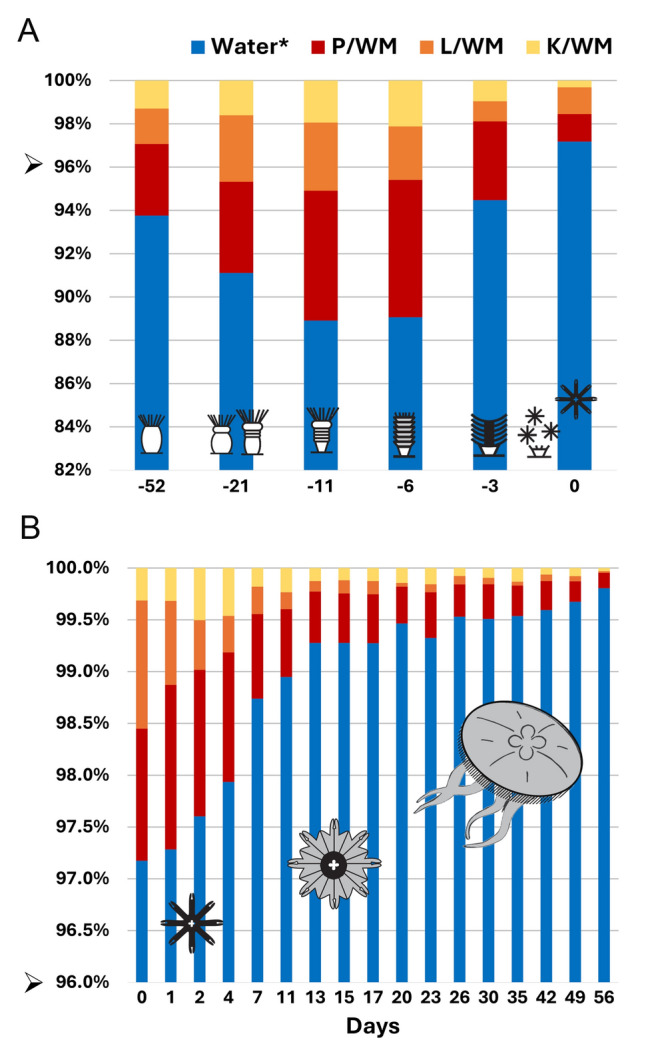
Stacked bar chart depicting the relative composition of water content (water*), protein (P), lipid (L), and carbohydrate (K) on each measurement day throughout the experiment. The charts are scaled to 100% to illustrate the proportional distribution of each component. Note that the y-axis does not start at zero to emphasize variations in composition. **A**: strobilation. **B**: ephyra-to-medusa transition. Arrowhead indicates the change in axis between A and B. Icons show the stage of the organisms and day 0 corresponds to ephyra release. The composition at day 0 (the day of the ephyra release) is present in both bar charts

The initial average polyp WM (2.28 mg) showed a notable increase during strobilation, reaching 3.34 mg in Stage I strobila (Fig. 2). This growth was primarily attributed to an increase P, L, and K (Fig. 3A). However, WM subsequently decreased to 2.24 mg in Stage III, only to experience a rapid surge to 4.86 mg in Stage IV, three days before ephyrae release. This increase in WM was mainly due to a rise in non-PLK components, particularly water content. Consequently, the WM of the Stage IV strobila was double that of the initial WM of the polyp (Supplementary Table S1). Throughout the ephyra-to-medusa transition, WM increased exponentially (Fig. 2). In fact, in the first two days post-release, the ephyra stage showed no significant change in WM.

Grouping composition results by life stage revealed that some data did not meet the normality and variance homogeneity requirements for parametric tests (Fig. 1). Consequently, post hoc pairwise comparisons were conducted using the Conover–Iman test whenever the Kruskal–Wallis test detected significant differences (*P* < 0.05) between groups (Supplementary Table S2). Significant differences (*P* < 0.05) in water content (water*) was observed across all life stages, except between sessile stages, where water* remained stable and lower than in the planktonic stages. Similarly, significant differences (*P* < 0.05) were noted in P/WM, L/WM, and K/WM among life stages. To mitigate the influence of water* fluctuations, organic components were also normalized by the total P + L + K (PLK).

This analysis showed no significant differences in P/PLK and L/PLK among polyps, and ephyrae, nor among metaephyrae and medusae. However, significant differences emerged when comparing polyps, and ephyrae to metaephyrae and medusae. K/PLK varied significantly only between ephyrae and metaephyrae. In this sense, water* increased as ephyrae differentiated from the sessile stages. Following this, significant alterations in P, L, and K content, accompanied the transition to metaephyra.

Daily data analysis revealed specific changes not apparent when grouped by life stages. In sessile stages, P, L, or K collectively constituted a higher percentage of WM, with water* ranging between 89 and 94% (Supplementary Tables S1, S2). In contrast, in planktonic stages, water* constituted between 97 and 99.5% (Supplementary Tables S1, S2). A substantial increase in water* was evident spanning from Stage (III, day -6), stage (IV, day −3), and the ephyra (Fig. 3A). Between day 0 and day 13 of ephyra–to–medusa transition, water* increased substantially. From strobila stage III (day –6) to metaephyra, in less than 20 days, the water content changed from around 89% to around 99% (Fig. 3B). Therefore, changes in WM were closely associated with variations in water*.

During the first stages of strobila, the percentage of all components (P, L, and K) increased. However, their proportion decreased as the water* component took on more significance, particularly at Stage IV (day –3), just three days before ephyra release (Fig. 3A). Notably, the component exhibiting the most pronounced changes was L, which experienced an increase during strobilation and underwent a decline leading up to strobila Stage IV (Fig. 3A). Conversely, in the ephyrae composition on the day of release, the concentrations of P and L were nearly identical (Supplementary Tables S1, S2), indicating that the ephyrae had the highest L percentage of organic components (L/PLK). L, however, decreased rapidly during the transition from ephyra to metaephyra and remained low in both metaephyra and medusa stages (Fig. 3B).

The analysis of respiratory and excretory metabolism revealed distinct patterns. Notably, the R, ETS, and IDH showed a high correlation WM and P (R^2^ > 0.97). Differences between stages revealed that WM-specific metabolism was most pronounced in sessile life stages. In planktonic life stages, ephyrae exhibited higher WM-specific metabolism compared to medusae. These WM-specific observations may be influenced by the changes in water* (Fig. 3; Supplementary Table S3).

**Fig. 4:**
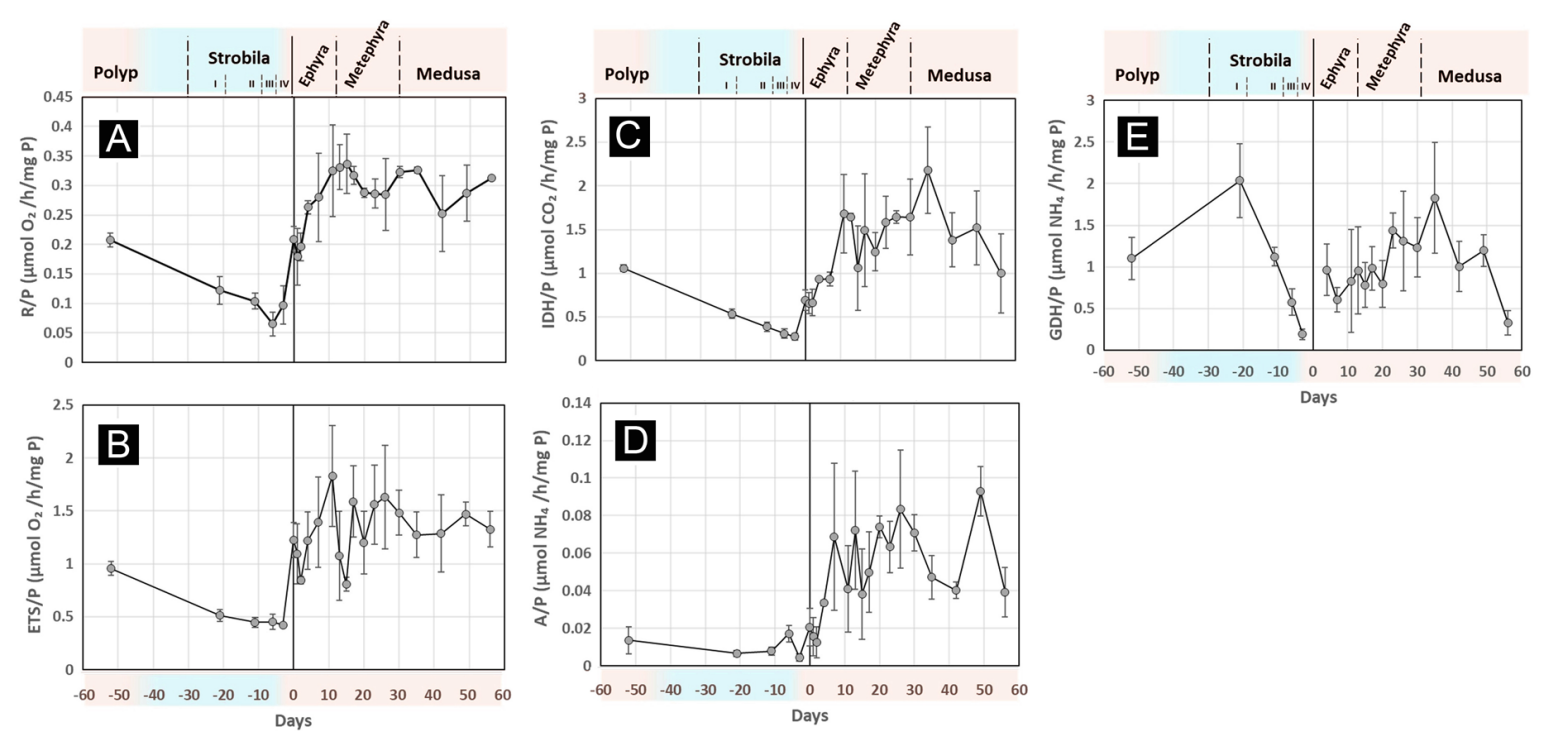
Time-series of the metabolic average results on each day of an experiment during strobilation. Metabolic activity per hour and per mg of protein for **A**: physiological respiration (R), **B**: electron-transport system respiratory activity (ETS), **C**: isocitrate dehydrogenase respiratory activity (IDH), **D**: physiological ammonium excretion (A), **E**: glutamate dehydrogenase excretory activity (GDH). From day -52 to day 0 corresponds to strobilation polyps transitioning to strobila and ephyra release on day 0. Measurements after and including day 0 correspond to ephyra transition to medusae. The top axis shows the life stages of the samples. Error bars are standard deviation. Each point is n = 9. GDH data below the detection limit at days 0, 1, and 2 are absent in the figure. The results of all stages were obtained at 20 ºC (in light orange) except the strobila stages (in light blue) which were obtained at their in situ temperature (10 ºC)

P-specific respiratory metabolism (R/P, Φ/P, IDH/P) and excretory metabolism (A/P, GDH/P) during metamorphosis are shown in Fig. 4. Statistical comparisons based on the life stage delineations shown in Fig. 1 indicated that some results did not meet the normality and variance homogeneity assumptions required for parametric tests. Therefore, post hoc pairwise comparisons were performed using the Conover–Iman test when the Kruskal–Wallis test detected significant differences (*P* < 0.05) among groups, as detailed in Supplementary Table S4.

Ephyrae in the first days post-release showed respiratory metabolism comparable to that of pre-strobilation polyps. There were no significant differences in R/P or ETS/P between polyps and ephyrae (Supplementary Table S4). As ephyra transitioned to metaephyra, P-specific respiratory metabolism increased rapidly and stabilized by day 11, remaining consistent in the medusa stage (Fig. 4A-C). Although metaephyra and medusa showed no significant differences in R/P, ETS/P, or IDH/P compared to each other, these parameters differed significantly from those in earlier life stages (Supplementary Table S4). P-specific metabolism in the medusa stage was approximately 1.5 times that in polyps (Supplementary Tables S3, S4).

P-specific ammonia excretion (A/P) remained relatively constant during the initial stages of strobilation but doubled by Stage III (day -6) and then halved by Stage IV (day -3). During the transition from ephyra to medusa, A/P experienced a significant increase, averaging more than three times that observed in ephyrae or polyps. A/P was significantly lower for polyps compared to the planktonic life stages (Fig. 4D; Supplementary Table S4).

The parameter with the highest variability was GDH/P, which may account for the lack of significant differences between life stages. GDH/P peaked in strobila Stage I and then gradually decreased through Stage IV. During ephyra release, GDH/P was either barely detected or below the detection limit of the technique for the first few days. During the transition from ephyra to medusa, GDH was not detected until day 4, after which it steadily increased to levels similar to those in polyps. However, during the medusa stage, a decrease in GDH/P was observed, with the final measurement being less than half of the GDH/P observed in polyps (Fig. 4E; Supplementary Table S4).

The ratio between the physiological measurements (R, A) and their enzymatic equivalents (Φ, GDH) was relatively low (Supplementary Table S4). R/Φ remained constant at approximately 22 ± 5%. A/GDH was lower in sessile life stages (1.5 ± 1.1%) and could not be calculated on days 0, 1, and 2 post-ephyra release due to the low GDH activity. In contrast, during the planktonic life stages, the A/GDH ratio was nearly four times higher, averaging 5.6 ± 2%.

## Discussion

Numerous studies have examined the relative composition of the main proximate components of *A. aurita*, generally medusae, highlighting its high-water content, rich P content, and low levels of K and L (Abdullah et al. 2015; Arai 1997; Båmstedt et al. 1994; Khong et al. 2016; Larson 1987; Lucas 1994; Matsakis and Conover 1991; Schneider 1989). The findings of the present study are consistent with these observations, reinforcing the understanding of *A. aurita*’s biochemical composition across different life stages.

**Fig. 5:**
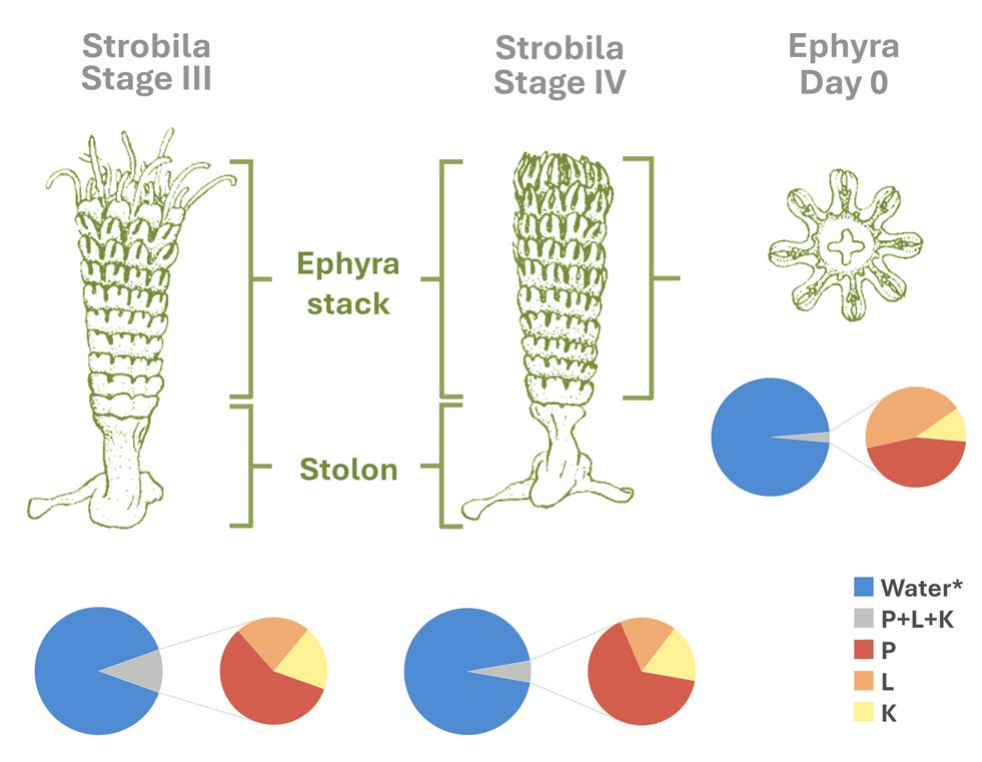
Anatomical diagrams based on the observations of Kato et al. (1973) alongside labels of the sections of the strobila that may showcase different compositions, and pie charts depicting the average water*, P, L, and K composition of each stage

Polyps in particular may accumulate more energy-rich compounds as an adaptive strategy to their sessile lifestyle, limited prey capture surface, thereby supporting the energetically demanding process of strobilation. Aligned with this idea, our results show that L and K concentrations peak during strobilation but decline with the cessation of ingestion between days –6 and –3 before the ephyra release, coupled with the loss of tentacles (Russell 1970) which marks the onset of significant anatomical and compositional changes (Figs. 1, 5; Supplementary Table S1). Similarly, González-Valdovinos et al. (2019) studied the metamorphosis of the cannonball jellyfish (*Stomolophus meleagris*) and found that polyps exhibit better carbohydrate hydrolysis. They attributed this to the high digestive activity of amylase and aminopeptidase in polyps, which provides an advantage, because they are sessile and have lower chances of encountering food.

Our findings also reveal significant shifts in biochemical composition across the life cycle, particularly during strobilation and ephyra-to-medusa transition. As hypothesized, notable increases in P, L, and K were observed during strobilation, reflecting the high energy demands of this stage. This is consistent with previous studies in which it was shown that L and K are the main sources of biological energy during metamorphosis (Agrell and Lundquist 1973; Beenakkers et al. 1981; Lucas 1994; Nestel et al. 2003; O’Boyle and Beamish 1977).

Lucas (1994) reported high L content in ephyrae. Even though the lipid result for the youngest ephyrae was missing, their study suggested that initial ephyrae could contain a lipid concentration equivalent to that of protein. Lucas’s hypothesis is supported by our results, where L was equivalent to P at the moment of ephyrae release on day 0 (Fig. 3A, B). These details of strobilae and ephyrae composition during strobilation could have implications for their nutritional value. Stenvers et al. (2020) reported on average higher concentrations of essential fatty acids in ephyrae, potentially making them a valuable food source for specific predators (Östman 1997; Takao et al. 2014).

The energy reserves in ephyrae are particularly crucial, given their high energy requirements (Lucas et al. 2012). Therefore, it is also possible that, aside from energy storage, the initial high lipid content may aid in buoyancy and swimming (Kattner et al. 2007). For the ephyra life stage, the combination of these functions, i.e., energy storage and a buoyancy enhancement, could be critical during the transitional period as the bell fills out to a full umbrella (von Montfort et al. 2023). Our results indicate that WM-specific energy reserves are sufficient to meet these demands until the end of ephyra stage (day 7). Previous studies have shown that ephyrae can survive for up to 33 and 60 days under starvation conditions, at temperatures ranging from 9 to 15 ºC (Fu and Uye 2021). Furthermore, during metamorphosis, the development of the mesoglea—a gelatinous, acellular tissue that forms the structural framework of the medusa life stage—provides another critical insight into the biochemical shifts observed in this study. The mesoglea is primarily composed of water (up to 99% in some species) along with proteins such as mesogloein which provide structural support (Matveev et al. 2012). Kotova et al. (2015) emphasized that the principal difference between the polyp, strobila, ephyra, and medusa life stages is the mesoglea.

The transition from polyps to strobilae at lower temperatures increases individual mass (Wang et al. 2018; Fig. 2; Supplementary Table S1), largely due to accumulation of water* by Stage IV (Fig. 5). In this context, water content increases as ephyrae differentiates from the sessile stages. This is followed by significant changes in P, L, and K content, during the transition to metaephyra, reflecting extended morphological adjustments. As ephyrae mature to metaephyrae, water content continues to rise, further illustrating the dynamic biochemical and structural changes associated with *Aurelia aurita*’s metamorphosis toward the medusa life stage.

Metabolic studies of metamorphosis in cnidarians, particularly scyphozoans, are scarce. Gambill and Peck (2014) concluded in their study that mass-specific respiration rates of *A. aurita* polyps, ephyrae, and medusae are similar. However, their results were mainly based on previously published single life-stage studies. Here, we measured *A. aurita’s* metabolic rates during life-stage transitions from polyp to medusa and showed that the R-to-P-specific (Supplementary Table S4) and R-to-WM-specific results (Supplementary Table S3) differ among life stages (*P* < 0.05),

When comparing our respiratory data, converted to µL O_2_ (1 µmol O_2_ = 22.391 µL O_2_) to existing literature (Han et al. 2012; Iguchi et al. 2017; Møller and Riisgård 2007; Shimauchi and Uye 2007; Uye and Shimauchi 2005), they align well within the reported range. Moreover, the WM-specific respiration rates for the planktonic life stages, ranging between 10.5 in medusa and 75.7 µL O_2_ h^−1^ g WM^−1^ in 4-day-old ephyra (Supplementary Table S3), fall within the wide range of respiratory measurements reported in the literature, according to those compiled by Bondyale-Juez et al. (2022), i.e., 2.96—180 µL O_2_ h^−1^ g WM^−1^.

Few studies have centered on ephyrae respiration, and our results are slightly below those of 108.2 µL O_2_ h^−1^ g WM^−1^ reported by Mangum et al. (1972). Roberts (1957) noted that smaller organisms typically have higher WM-specific respiratory activities, and Kinoshita et al. (1997) suggested that ephyra and metaephyra require more activity to swim due to their morphology. Our R-to-WM-specific results seem to support these suggestions; however, the significant change in water content complicates comparison, and R-to-P-specific results do not show higher activity in younger stages (Fig. 4; Supplementary Table S4).

Regarding polyps, our pre–strobilation results (151.7 ± 40.7 µL O_2_ h^−1^ g WM^−1^, Supplementary Table S4) are above those reported by Purcell et al. (2019) (15 – 141 µL O_2_ h^−1^ g WM^−1^). However, it is important to point out that none of the polyps compiled by Purcell et al. (2019) were fed daily. Additionally, no previous respiration measurements in strobilae or during strobilation were found.

The excretory metabolism behaved very differently. Cnidarians, like most aquatic animals, excrete primarily ammonium (NH_4_^+^) (Hubot et al. 2021; Wright 1995). P-specific ammonium excretion (A) was higher in the medusa life-stage. Ingestion increased throughout the planktonic life stage, increasing excretion rates and rising excretory metabolic activity. Only a few NH_4_^+^ excretion experiments have been carried out in *A. aurita* (Hubot et al. 2021; Nemazie et al. 1993; Schneider 1989; Shimauchi and Uye 2007). This is the first study to measure NH_4_^+^ excretion in *A. aurita* from the polyp to medusa life stage. NH_4_^+^ is largely formed and excreted as a by-product of amino-acid catabolism (Nelson and Cox 2008; Weihrauch and Allen 2018). Lower NH_4_^+^ excretion rates imply lower rates of amino-acid catabolism. Amino acids cannot be stored in animal tissue, so their excess, i.e., those that cannot be used in P production, are catabolized producing NH_4_^+^, and then excreted. In general, our results for the planktonic stage of *A. aurita* (A = 0.072 – 0.51 µmol NH_4_^+^ h^−1^ g WM^−1^ and 0.012 – 0.093 µmol NH_4_^+^ h^−1^ mg P^−1^; Supplementary Tables S3, S4) fall above previous excretion measurements reported in the literature (A = 0.021 – 0.071 µmol NH_4_^+^ h^−1^ g WM^−1^ or 0.005 – 0.011 µmol NH_4_^+^ h^−1^ mg P^−1^, Biggs 1977; Hubot et al. 2021; Muscatine and Marian 1982; Schneider 1989; Shimauchi and Uye 2007). The explanation for this discrepancy could be related to the daily feeding regime, the period left between feeding and before measuring excretion (Hubot et al. 2021), or the fact that some values in the literature came from day-long measurements. No previous published excretion measurements in polyps or strobilae were found.

Enzymes, as indices in biological oceanography, could be useful tools in metamorphosis studies (García-Esquivel et al. 2001). Theoretically, ETS, IDH, and GDH activities could provide quantitative information on the maximum rates attainable in the respiratory ETS and the TCA Cycle. In addition, if these activities were incorporated in the enzyme kinetic model used by Aguiar-González et al. (2012), Fernández-Urruzola et al. (2016a, b), and Osma et al. (2016), they should serve as reliable proxies of rates of oxygen consumption and ammonium excretion in jellyfish. In any case, these activities should provide a basis for comparison with the metabolic rates that were concurrently measured in this study (RO_2_ and NH_4_^+^ excretion). Studies of enzymatic activity in Scyphozoa, however, are rare (Aljbour et al. 2017; Iguchi et al. 2017; King and Packard 1975; Purcell et al. 2019; Thuesen and Childress 1994).

The maximal activity of the respiratory enzymes ETS and IDH shifted during metamorphosis, thereby showcasing changes in their concentration (Fig. 4). These enzymes are located in the mitochondria and one potential explanation is that the number of mitochondria varies during metamorphosis (Locke 1981). Mitochondria need to be enumerated to test this hypothesis. Different enzyme concentrations through metamorphosis have also been observed in weight-specific ETS of Pacific oyster (*Crassostrea gigas*) (Garcıa-Esquivel et al. 2001). They showed that ETS concentrations differed within 24 days of post-settlement and displayed an annual variability. An alternative explanation for mitochondrial activity decline could be changes in the electron-transport system composition during metamorphosis (Chamberlin 2007) or apoptosis. Furthermore, programmed cell death during metamorphosis has been investigated in coral cnidarian *Hydractinia* (Seipp et al. 2001; Wittig et al. 2011) and in metamorphosing *A. aurita* planula larvae (Yuan et al. 2008).

The R/Φ ratio can provide insight into the stress condition of an organism (St-Amand et al. 1999). It reflects how much of an organism’s energy-transforming potential is being utilized. In our study, the R/Φ ratio was low throughout life-stage transitions. In other words, all life stages in these laboratory-raised conditions had a low basal metabolism compared to their potential (Supplementary Table S4). Purcell et al. (2019) reported that *A. aurita* polyps have a low R/Φ ratio independent of food supply and starvation. These findings were unexpected as previous studies had suggested that diet affects the metabolism in marine organisms (Hernández-León and Gómez 1996; Martínez et al. 2010; Romero-Kutzner et al. 2015). Thus, the R/Φ ratio throughout *A. aurita’s* life cycle (from polyp to medusae) may reflect consolidated transitions, since, despite shifts in biochemical composition and physiological rates, it did not deviate significantly from basal metabolism. However, it is important to note that this study was conducted under controlled laboratory conditions, and the findings may not be fully extrapolatable to the natural environment.

Likewise, the enzyme GDH is an important enzymatic link between amino acids and the tricarboxylic acid cycle (TCA) (Nestel et al. 2003). Most amino acids are first transaminated to form ionic glutamate. Glutamate is then enzymatically deaminated by GDH to form NH_4_^+^ and alpha-ketoglutarate. Alex (1971) showed that GDH increases its affinity (K_M_: Michaelis–Menten constant) for coenzymes during tadpole-to-frog metamorphosis. This affinity favors the production of NH_4_^+^ and alpha-ketoglutarate. In this sense, a change in affinity for coenzymes could be an additional explanation of why different enzyme concentrations are present in *A. aurita*’s life-stage transitions (Fig. 4E).

The GDH/NH_4_^+^ excretion ratio in ephyrae to medusae was 24.4 ± 14.8 (n = 107). This ratio is consistent with the observations by other investigators (Bidigare and King 1981; Bidigare et al. 1982; Fernández-Urruzola et al. 2011; Hernandez-Leon and Torres 1997). One possible explanation for the uncoupling between A and GDH (Fig. 4D, E) is that A measurements reflect time-dependent changes in NH_4_^+^ concentration in the incubation water, whereas nitrogenous components in the mucus of organisms may contribute to excretion observed (Hubot et al. 2022). Consequently, part of the difference between the A/P observed during the transitions from polyp to ephyra and metaephyra to medusa could be linked to nitrogenous compounds excreted through mucus production. This could also partially account for the discrepancy between A and the GDH activity. A was detected when GDH activity was lowest, and below our detection limit, particularly during the period when ingestion ceased, and L appeared to be the main energy source. In this context, it would be expected that GDH activity involved in protein catabolism would decrease. However, the response in A was not as clear. This could be due to other processes excreting N compounds besides traditional GDH-associated NH_4_^+^ excretion, such as the mentioned generation of mucus.

## Conclusions

The present study is the first to monitor both metabolic changes (respiration and excretion) and compositional changes (P, L, and K) throughout the metamorphosis of *Aurelia aurita*, from polyp to medusa, including strobilation and planktonic transformation across several stages: strobila, ephyra, metaephyra, and medusa. Our findings reveal significant shifts in both metabolism and biochemical composition, providing novel insights into the dynamic processes during the life cycle of *A. aurita*.

A key observation is the dramatic change in water content throughout metamorphosis. This increase in water content was a major driver behind the exponential rise in WM as the organism grows toward juvenile medusae. Additionally, the observed shifts in metabolism and biochemical composition underscore their potential influence on nutrient cycling, trophic interactions, and the resilience of *A. aurita* to environmental stressors.

These metabolic transitions may also serve as indicators of vulnerabilities to environmental change, emphasizing the importance of monitoring multiple life stages in future ecophysiological studies. However, as our understanding of *A. aurita*’s metamorphosis continues to develop, further research is necessary to explore the underlying mechanisms and their ecological implications.

## Supplementary Information

Below is the link to the electronic supplementary material.

Supplementary file1 (RAR 63 KB)

## Data Availability

All data supporting the findings of this study are available within the paper and its Supplementary Information.
